# The β6/β7 region of the Hsp70 substrate-binding domain mediates heat-shock response and prion propagation

**DOI:** 10.1007/s00018-017-2698-3

**Published:** 2017-11-09

**Authors:** Linan Xu, Weibin Gong, Sarah A. Cusack, Huiwen Wu, Harriët M. Loovers, Hong Zhang, Sarah Perrett, Gary W. Jones

**Affiliations:** 1 Department of Biology, Maynooth University, Maynooth, Co. Kildare, Ireland; 2 National Laboratory of Biomacromolecules, CAS Center for Excellence in Biomacromolecules, Institute of Biophysics, Chinese Academy of Sciences, Beijing, 100101 China; 3 University of the Chinese Academy of Sciences, Beijing, 100049 China; 4 Present Address: Department of Clinical Chemistry, Certe, Groningen, The Netherlands; 5 Present Address: Centre for Biomedical Science Research, School of Clinical and Applied, Leeds Beckett University, Portland Building, City Campus, Leeds, LS1 3HE UK

**Keywords:** Hsp70, Substrate-binding domain, Allosteric regulation, Prion, Heat shock

## Abstract

Hsp70 is a highly conserved chaperone that in addition to providing essential cellular functions and aiding in cell survival following exposure to a variety of stresses is also a key modulator of prion propagation. Hsp70 is composed of a nucleotide-binding domain (NBD) and substrate-binding domain (SBD). The key functions of Hsp70 are tightly regulated through an allosteric communication network that coordinates ATPase activity with substrate-binding activity. How Hsp70 conformational changes relate to functional change that results in heat shock and prion-related phenotypes is poorly understood. Here, we utilised the yeast [*PSI* ^+^] system, coupled with SBD-targeted mutagenesis, to investigate how allosteric changes within key structural regions of the Hsp70 SBD result in functional changes in the protein that translate to phenotypic defects in prion propagation and ability to grow at elevated temperatures. We find that variants mutated within the β6 and β7 region of the SBD are defective in prion propagation and heat-shock phenotypes, due to conformational changes within the SBD. Structural analysis of the mutants identifies a potential NBD:SBD interface and key residues that may play important roles in signal transduction between domains. As a consequence of disrupting the β6/β7 region and the SBD overall, Hsp70 exhibits a variety of functional changes including dysregulation of ATPase activity, reduction in ability to refold proteins and changes to interaction affinity with specific co-chaperones and protein substrates. Our findings relate specific structural changes in Hsp70 to specific changes in functional properties that underpin important phenotypic changes in vivo. A thorough understanding of the molecular mechanisms of Hsp70 regulation and how specific modifications result in phenotypic change is essential for the development of new drugs targeting Hsp70 for therapeutic purposes.

**Electronic supplementary material:**

The online version of this article (10.1007/s00018-017-2698-3) contains supplementary material, which is available to authorized users.

## Introduction

Heat-shock proteins are critical components of in vivo defence mechanisms against physical and chemical stresses. Heat shock, for example, can cause the unfolding and misfolding of proteins, as well as a loss of membrane integrity. Without efficient intracellular defence mechanisms, changes in membrane status and disruptions to global protein function inevitably cause cell death [1, 2]. Under mild heat shock, heat-shock response elements (HSEs, 5′-nAGAAnnTTCTn-3′), and stress responsive elements (STREs) govern the expression of stress-response genes and heat-shock proteins such as Hsp70, through the binding and activation of heat-shock factor (HSF) [3, 4]. Hsp70 is a ubiquitous and highly conserved 70 kDa protein that assists in protein folding and refolding, and protects cells from a variety of stresses [5]. A plethora of cytoplasmic chaperones such as Hsp110, Hsp90, Hsp40, small Hsps, and mitochondrial chaperones such as Hsp10 and Hsp60 are also induced under heat shock [6].

The vast majority of information on the mechanism of Hsp70 regulation has been acquired through studies of the *Escherichia coli* Hsp70 protein DnaK [7–13]. In *E. coli,* the abundance and functionality of DnaK and chaperonin GroEL are enhanced following a heat shift from 37 to 43 °C [14]. Structurally, Hsp70 is comprised of two domains: a 45 kDa N-terminal nucleotide-binding domain (NBD) enriched in α-helices with weak ATP hydrolysis activity and a 25 kDa C-terminal substrate-binding domain (SBD) consisting of a β-sheet-rich substrate-binding cavity (SBDβ), with a flexible α-helical lid region (SBDα) that regulates access to the cavity, and a disordered tail region of approximately 30 residues [10, 15, 16]. There is bidirectional allosteric intramolecular regulation between the NBD and SBD. ATP binding and NBD hydrolysis have been shown to regulate substrate capture by the SBD, and interestingly, binding of substrate to the SBD increases ATP hydrolysis [10]. Comparing DnaK-ADP with DnaK-ATP indicates the ATP state to be more compact. In the ATP-bound state, the lid region is completely detached from the substrate-binding pocket and docked onto one side of the NBD with the linker region buried in the cleft of the NBD to facilitate SBD docking with another NBD region [8, 17]. Several residues in DnaK have been identified at this NBD:SBD interface: R151, R167, D326, D393, K414, and D481 [8, 9]. Importantly, the integrity of this interface is essential to allosteric regulation and intramolecular communication in Hsp70. In particular, residues D481 and K414 act as clamps, fixing the NBD:SBD interaction in the ATP-bound state and decreasing ATPase activity, without stimulation from substrate or co-chaperones such as Hsp40 [9]. Recently, residues D481 and L484 in the SBD of DnaK (homologous to D480 and L483 in yeast Ssa1) have been shown to play critical roles in the regulation of signal transduction from the SBD to the NBD [9]. However, it is unclear at the molecular and structural levels, how allosteric heat-shock proteins themselves respond to elevated temperatures.

Hsp70 is a potential drug target for a variety of neurodegenerative diseases that manifest as protein misfolding disorders, such as Parkinson’s, Alzheimer’s, Huntington’s, and prion diseases [5, 18–20]. Prions are self-perpetuating protein aggregates, and a variety of yeast proteins have been demonstrated to form prions [21–23]. The ability to switch from the native to prion form appears to functionally influence and regulate normal biological cellular processes [24]. The [*PSI* ^+^] prion state has been at the forefront of utilising yeast as a model for studying the cellular factors that regulate prion propagation. [*PSI* ^+^] is the prion form of Sup35, a translation termination factor in *Saccharomyces cerevisiae* [25]. The formation of self-templating aggregates by Sup35 causes reduced translation termination efficiency and increases read-through of nonsense mutations [26]. Studies in yeast have identified protein chaperones as the main cellular factors regulating prion propagation, particularly the Hsp104, Hsp70, and Hsp40 families [27–30]. Once a prion is established in vivo, Hsp70s play a central role in modifying and influencing the prion state.

There are two major cytosolic Hsp70 families (Ssa and Ssb) that influence [*PSI* ^+^] propagation [31–34]. The Hsp70-Ssa subfamily has four members (Ssa1–4) that play a crucial role in protein folding, translocation and refolding of denatured proteins, and at least one member is essential to maintain cell viability [35, 36]. High levels of Ssa1 or Ssb1 can cure weak [*PSI* ^+^], as do high levels of the Hsp40s, Sis1, and Ydj1; however, strong [*PSI* ^+^] variants are largely unaffected by such treatments [37, 38]. Specific partner Hsp40s can stimulate ATPase activity and deliver substrates to Hsp70, while nucleotide exchange factors (NEFs) regulate the exchange of ADP for ATP and the re-binding of Hsp70 to substrate [39].

A variety of genetic and biochemical studies in yeast have identified many mutations of Hsp70 (Ssa1) that impair propagation of the yeast prions [*PSI* ^+^] or influence cell growth activity, the majority of which are located within the ATPase domain [28, 40, 41]. The first and most well characterized mutant—the *SSA1*–*21* variant—possesses an L483W change located within the SBD [34]. This L483W mutation impairs [*PSI* ^+^] propagation by reducing the number of prion “seeds” [34, 42]. A range of second-site suppressors of L483W, which restore [*PSI* ^+^] propagation, were identified in regions that influence substrate-binding efficiency [40]. In addition, deletion of Hsp70-Ssa NEFs Fes1 or Sse1 in combination with L483W improves [*PSI* ^+^] propagation [33]. This suggests that L483W causes an enhanced substrate-binding activity for Hsp70, a hypothesis supported by steered molecular dynamics simulations [43]. Preliminary biochemical analysis of L483W suggested that this mutation weakened substrate-binding activity of a small peptide substrate [44]. Reasons for this apparent discrepancy could be due to differences in the peptide substrate used in biochemical and computational studies, or simply due to the difficulties in experimentally defining this precise activity from a biochemical perspective [43]. Of particular note also is that many of the second-site suppressor mutations of L483W are located within the NBD, which suggests that impairment of prion propagation in the *SSA1*–*21* mutant may be related to inter-domain communication [40].

Although several prion-impairing Hsp70-Ssa mutants have been characterized extensively genetically, structural and functional analysis is notably lacking. Therefore, it remains unclear what specific functional changes occur within the Hsp70 SBD that result in impairment of prion propagation in yeast. In this study, we identify and characterize a new Hsp70-Ssa SBD mutant (F475S) that is severely impaired in both heat shock and prion propagation, and in combination with structural analysis of L483W, we identify a crucial β6–β7 region in the SBD that is important in substrate-binding and dictating the ability to impair prion propagation in yeast. In addition, structural and genetic studies on L483W allow us to identify residues at the potential interface between the NBD and SBD in yeast Ssa1. These critical SBD residues may play key roles in signal transduction between domains and regulate Hsp70 function in propagating prions and heat-shock response in vivo.

## Results

### SBDβ mutations alter [*PSI*^+^] propagation and heat-shock response

**Fig. 1:**
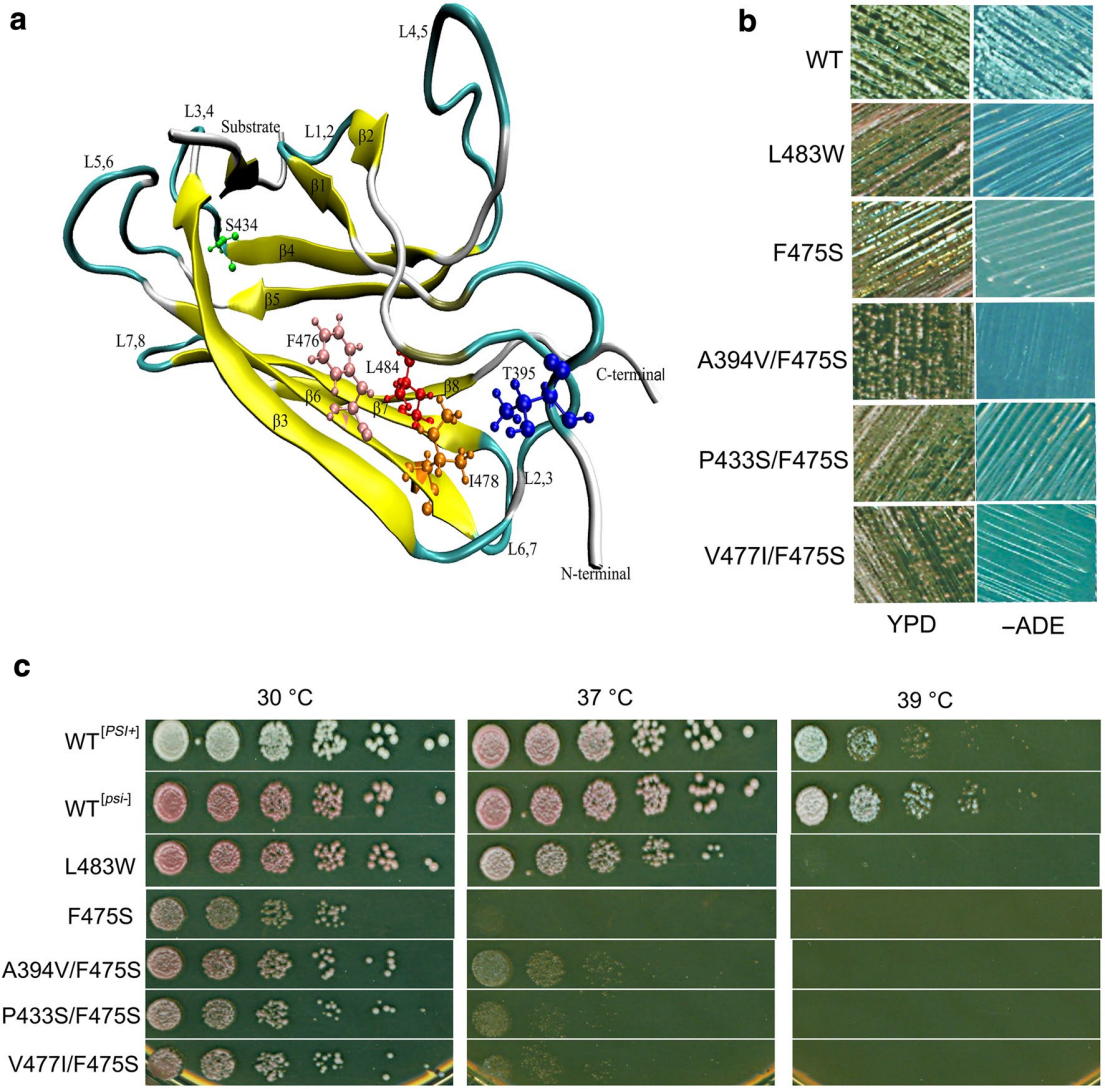
SBDβ mutations alter thermotolerance and [*PSI* ^+^] propagation. **a** Locations of SBD mutants on the DnaK structure. Ribbon schematic representations of the Protein Data Bank structure 1Q5L. The DnaK amino acid residue numbers are homologous to those of the Ssa1 mutations assessed. **b** Assessment of [*PSI* ^+^] propagation. [*PSI* ^+^] propagation assay at RT. Single colonies were streaked on YPD and −ADE plates which were then incubated at RT for 5–7 days. [*psi* ^−^] cells were red colonies on YPD and unable to grow on−ADE plates; [*PSI* ^+^] cells were white colonies on YPD and viable on −ADE plates. **c** Growth assay of SBD mutants at elevated temperatures. Fresh cultures were spotted onto YPD after a 1/5 serial dilution. Plates were incubated at 30 °C for 2 days

Yeast is a well-established model to investigate prion propagation, which can be easily monitored utilising colony colour and growth assays on selective media. [*PSI* ^+^] cells are white colonies on YPD and grow well on−ADE, and in contrast, [*psi* ^−^] cells are red and do not grow on−ADE. In this study, a modified random mutagenesis strategy based on colony colour was utilised to screen for SBD-specific mutants that alter [*PSI* ^+^] propagation. The screen identified four novel SBD mutations, V439I, F475S, M515I, and S545F, and two previously characterized mutants G481D [28] and L483W (Fig. 1a) [33, 43, 44]. As previously reported, the L483W variant is [*psi* ^−^] when the only Ssa source in cells, but grows well at 30 and 37 °C [34]. As shown here, F475S exhibits prion-impairing effects and is also temperature sensitive (ts) for growth (F475S and L483W are shown in Fig. 1b, c, respectively). In addition, L483W was ts at 39 °C (Fig. 1c) which was not previously tested. Given that the significant phenotypic effects of F475S upon both prion propagation and growth at elevated temperature, we focused on this mutant in the first instance for further analysis. Using random mutagenesis, we identified three second-site suppressors of F475S (A394V, P433S, and V477I). These mutations were modelled onto the structure of DnaK (Fig. 1a). None of the three mutants restored [*PSI* ^+^] propagation, but rescued temperature sensitivity of F475S to varying degrees (Fig. 1b, c).

The prion and ts phenotypes exhibited by F475S and L483W strains indicated that both are intrinsically linked to fundamental changes in Hsp70 activity. However, the ability to obtain partial suppression of the ts phenotype by second-site suppressors of F475S without any recovery of prion propagation highlights a significant difference between these two Hsp70 functional outputs.

### Amino acid substitutions at residues 475 or 483 disrupt conformation of SBDβ and cause functional instability

**Fig. 2:**
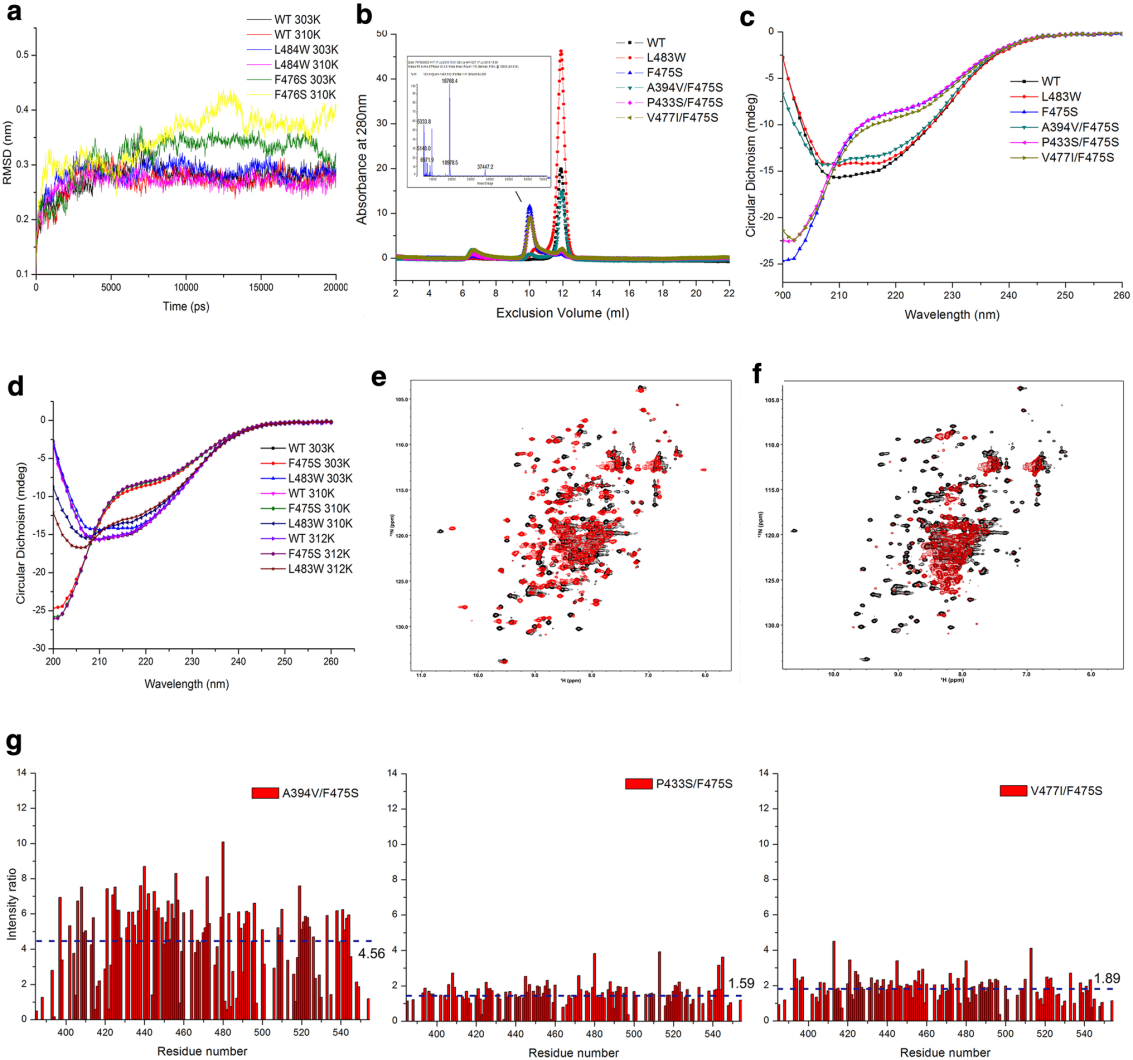
Amino acid substitutions at residues 475 and 483 alter Hsp70 SBDβ conformation and stability. **a** RMSDs as a function of the simulation time. RMSDs were calculated using the g_rms program based on the Cα atoms of the amino acid backbone of the DnaK protein. **b** Pattern of Ssa1 SBD truncation proteins. Size exclusion chromatography (SEC) was performed at RT; molecular weight of F475S truncation protein was assessed by MALDI-TOF (insert). **c** CD spectroscopy of SBD truncation proteins at 30 °C. **d** CD spectroscopy of SBD truncation proteins at elevated temperatures (37 and 39 °C). The temperature was precisely controlled using a Peltier device. **e** 2D ^1^H–^15^N HSQC spectra of WT (black) and L483W (red) at 30 °C. **f** 2D ^1^H–^15^N HSQC spectra of WT (black) and F475S (red) at 30 °C. **g** Intensity ratio of second-site suppressors. Spectra of SBD truncation proteins containing F475S and second-site suppressors were acquired at 30 °C. Residue arrangement was based on the residue arrangement of WT. Intensity was calculated under the same concentration and display levels. The values and dotted line indicate the mean intensity ratio for each mutant

Given the ts phenotypes, we hypothesized that F475S and L483W significantly alter the structure and function of the SBD at higher temperatures. To gain initial structural insight, we resorted to a previously utilised MD simulation based on DnaK modelling [43]. Following simulations, the structural stability of mutants was assessed by calculation of the root-mean-square deviation (RMSD) of the protein Cα atoms. The RMSD profiles indicated that the F476S (F476S in DnaK is an equal of F475S in Ssa1) altered the structural stability of the DnaK SBD at 30, 37, and 39 °C, but L484W (L484W in DnaK equates to L483W in Ssa1) only disturbed the SBD at 39 °C and with a much-reduced effect compared to F476S (Fig. 2a; Fig. S1), which suggests that they may cause temperature sensitivity by destabilizing the SBD.

To assess structural effects upon the SBD further, we purified Ssa1 truncation proteins (residues 382–554) [51], harbouring engineered mutations, and assessed them biochemically and biophysically. F475S displayed a decreased elution volume compared to monomeric WT; while L483W and A394V/F475S both maintained the monomeric state of the SBD at RT (Fig. 2b). MALDI-TOF of the F475S truncation protein detected only one peak corresponding to the monomeric molecular weight (around 18.8 kDa; Fig. 2b, insert), suggesting that the decreased elution volume may be caused by unfolding of the F475S truncation protein, not dimerization or oligomerization. Indeed, F475S displayed a clearly reduced CD signal at 215–217 nm (β-sheet), 208 and 222 nm (α-helix), but increased CD signal below 200 nm (disordered structure), which suggests there were significant alterations in the secondary structure of the F475S truncation protein regardless of temperature (Fig. 2c, d), which is in agreement with the MD predictions and SEC observations (Fig. 2a, b). L483W showed minor changes compared to WT at 30 °C (Fig. 2c), but with temperature elevation, the α-helix and β-sheet content gradually decreased (Fig. 2d). Moreover, P433S/F475S and V477I/F475S displayed a slight change in CD signal around 200–205 nm, suggesting that the P433S and V477I may influence temperature sensitivity of F475S by maintaining the secondary structure of SBDβ to some extent (Fig. 2c). Notably, A394V/F475S had a CD spectrum much more similar to that of WT (Fig. 2c) consistent with the SEC results (Fig. 2b). Indeed, A394V was consistently the best suppressor of F475S in terms of both phenotypic and biochemical properties. Considering Ala394 is located in the linker between the NBD and SBD domains, we speculate that residue 394 not only influences the secondary structure of the SBDβ, but is also involved in inter-domain communication in full-length Ssa1.

The L483W mutation results in chemical shift perturbations of a number of ^1^H–^15^N signals, which can be attributed to introduction of the bulky aromatic ring of the tryptophan side chain and resultant conformational changes (Fig. 2e). On the other hand, L483W does not result in obvious signal intensity change, whereas the F475S mutation dramatically decreases the backbone ^1^H–^15^N HSQC signal intensities of the SBD, and only intensities of signals of disordered residues (7.8–8.4 ppm in the ^1^H dimension) remain clearly visible (Fig. 2f). The impact of the F475S mutation leading to disordered structure is consistent with the results described above (Fig. 2a–d). Importantly, A394V, P433S, and V477I all improved the NMR signal intensity of F475S at 30 °C (increasing 3.56-, 0.59-, and 0.89-fold respectively), with A394V again exhibiting the most dramatic effects (Fig. 2g).

Taken together, the data suggest that F475S significantly destabilizes SBDβ inducing unfolding, which cause the temperature sensitivity of cells. Second-site suppressors partially rescue the conformational changes to varying degrees. Comparing the heat shock and [*PSI* ^+^] phenotypes of all the mutants, it is clear that prion propagation is much more sensitive to perturbations in Hsp70 structure that result in altered or reduced function compared to essential roles for this ubiquitous chaperone.

### Structural changes induced in the β5/β7/β8 hydrophobic core

**Fig. 3:**
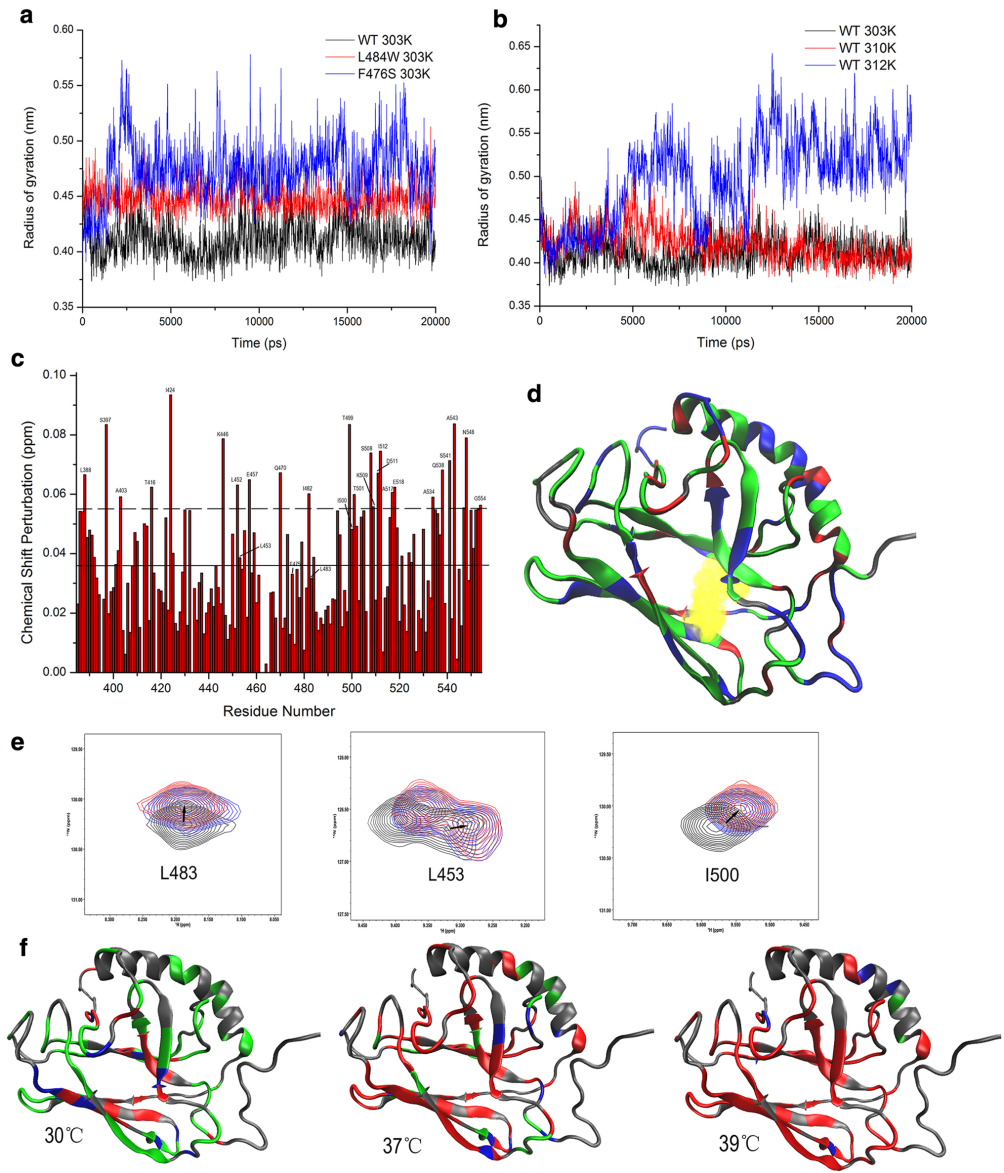
β5/β7/β8 hydrophobic core triggers the SBDβ allosteric network. **a** Radius of gyration of three highly conserved residues (Leu454, Leu484, and Ile501 in DnaK) as a function of simulation time at 30 °C for WT, F475S (F476S in DnaK) and L483W (L484 in DnaK). **b** Radius of gyration of three highly conserved residues (Leu454, Leu484, and Ile501 in DnaK) of WT as a function of simulation time at different temperatures. **c** CSPs histogram of WT between 30 and 39 °C. The solid line shows the average of CSPs; the dotted line shows the average plus SD. **d** Mapping chemical shift perturbations between 30 and 39 °C onto the DnaK structure (PDB:1BPR). Unassigned residues are showed in grey; CSP of residues less than average are shown in green; CSPs more than the average but less than average plus SD are showed in blue; significant CSPs (more than average plus SD) are shown in red. **e** Peak displacement pattern of three highly conserved residues in the hydrophobic core of WT at elevated temperatures; 30, 37, and 39 °C are represented black, blue, and red, respectively. **f** Residue perturbations of F475S compared to that of WT at 30, 37, and 39 °C on DnaK model. Grey residues that cannot be assigned in NMR spectra. Green: CSP is less than 0.5 peak width. Blue: CSP is between 0.5 and 1.0. Red: CSP is more than 1.0 peak width

Recently, it has been suggested that binding of the hydrophobic residues of peptide substrates requires an expansion of the substrate-binding pocket while in the ATP-bound state, leading to an overall reorganization of the SBDβ [9, 10], which may be trigged by the β5/β7/β8 hydrophobic core [13]. Based on the location of F475 and L483 (Fig. 1a), it might be expected that these mutations will destabilize SBDβ or alter the conformation of SBDβ by disrupting the hydrophobic core. As indicated from the results above, the SBDβ of F475S is indeed substantially disrupted. To investigate this further for the SBDβ of WT and L483W, we calculated the radius of gyration of three highly conserved residues (Leu454, Leu484, and Ile501 in DnaK) within the hydrophobic core using MDs. Figure 3a shows that L484W (L483W in Ssa1) and F476S (F475S in Ssa1) both have a loosened hydrophobic core at 30 °C. The loosening of the hydrophobic core also occurred in WT but only at 39 °C during simulations (Fig. 3b). Based on NMR chemical shift perturbation (CSP) of WT protein comparing 30 to 39 °C (Fig. 3c), we mapped shifted residues onto WT SBD truncation protein (Fig. 3d). The three hydrophobic core residues, Leu453, Leu483, and Ile500, displayed obvious CSPs (Fig. 3c, e), indicating that a variety of conformational changes are caused by elevated temperature. Indeed, the other residues next to those three hydrophobic core residues also induced significant CSPs (Leu452, Ile482, Thr499, and Thr501; Fig. 3c), which further supports the idea of a loosened hydrophobic core at elevated temperatures.

Due to dramatic signal attenuation, a complete backbone resonance assignment (> 90%) for the F475S mutant cannot be achieved. However, according to comparison of 2D ^1^H–^15^N HSQC spectra of F475S and WT, we can assign a substantial proportion of F475S signals, and calculated the CSPs for the F475S mutant (Fig. 3f), which clearly suggests perturbation of the hydrophobic core of the SBD at 30 °C, which is exacerbated with elevated temperature (Fig. 3f). It, therefore, appears that high temperature can induce an expansion of the hydrophobic core, and F475S and L483W substitutions accelerate this perturbation. Consequently, once triggered in the β5/β7/β8 hydrophobic core, the instability radiates to the whole SBD and perhaps then extends to the NBD.

### Disruption of SBDβ abolishes the SBD-dependent inhibition of ATP hydrolysis by the NBD and alters interactions with co-chaperones

The data presented thus far indicate substantial structural changes of SBDβ in mutants, even at permissive temperatures. The Ssa1 mutants may become less stable and could, therefore, alter ATPase regulation and affect the equilibrium between closed and open conformations. Such functional changes could be responsible for the in vivo temperature sensitivity and impairment of prion propagation phenotypes. To investigate this hypothesis, we assessed a series of biochemical characteristics of the full-length Ssa1 mutant proteins.

**Fig. 4:**
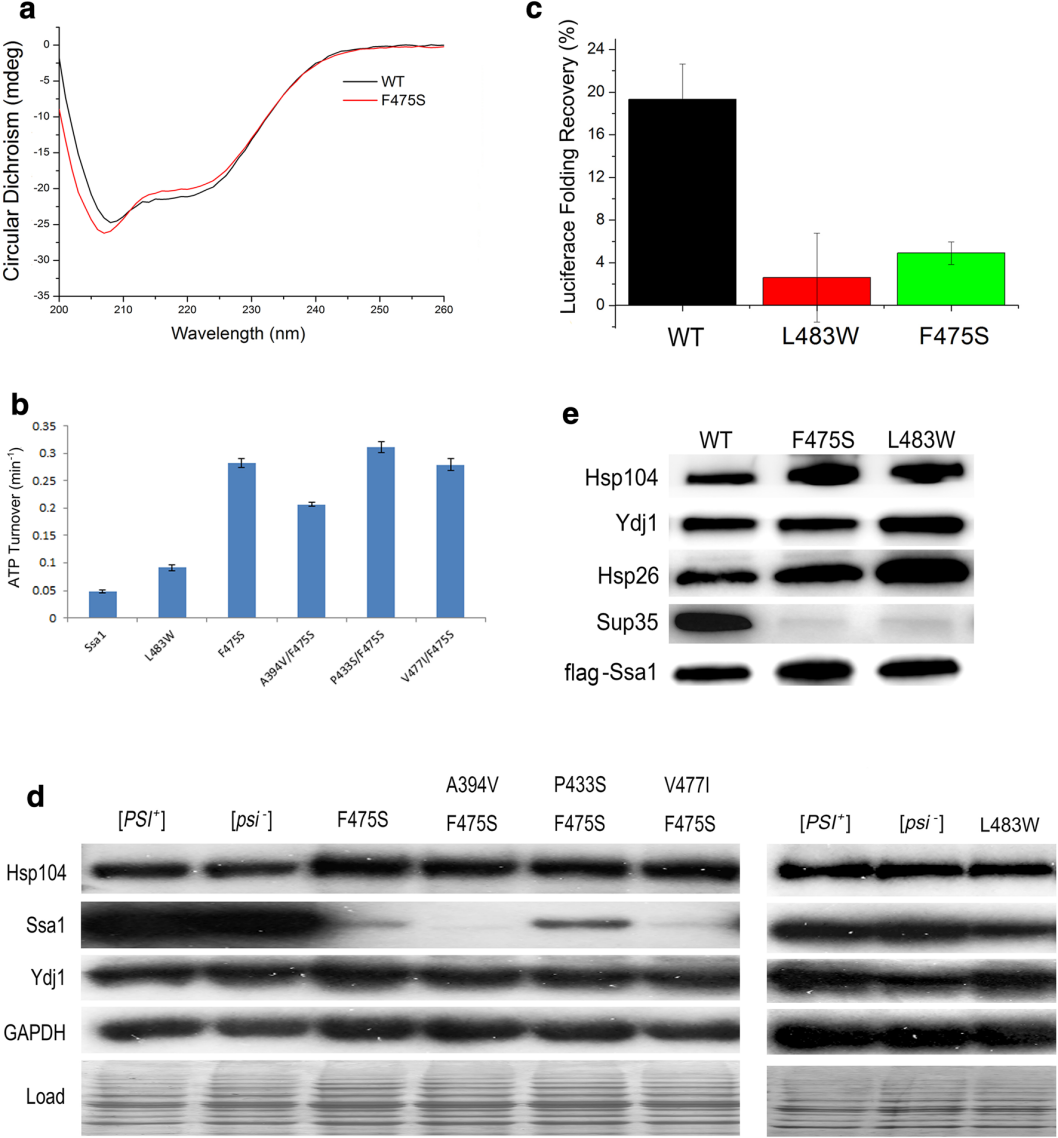
Disruption of SBD alters functions of Ssa1. **a** Secondary structure monitored by far-UV CD spectra for full-length Ssa1 at 30 °C. **b** ATPase activity of the full-length Ssa1 proteins. The unit of the ATP turnover rate is min^−1^. The values shown are the mean of four replicates from independent measurements and the error bars represent the standard deviation. **c** Luciferase refolding activity of F475S and L483W mutation yeast strains. Fresh cultures were shifted to 37 °C for 30 min before 45 °C denaturation for 1 h. Denatured luciferase cultures were recovered at 25 °C for a 1 h period. Cycloheximide was added to prevent protein synthesis during the recovery period. **d** Chaperone abundance of the Hsp70 machinery. Western blotting was performed to assess the expression levels of Hsp104, Ssa1, and Ydj1. GAPDH and a stained SDS-PAGE ran under the same conditions were used as loading controls. **e** F475S and L483W substitutions alter the Ssa1 interactions with clients. FLAG-tagged Ssa1 was pulled down from G402 cells and probed for Hsp104, Ydj1, Hsp26, and Sup35. FLAG-Ssa1 was used as loading control

CD spectra revealed that there were several differences in the secondary structure of full-length Ssa1 harbouring the F475S substitution, compared to wild-type protein. Briefly, CD signal differences are focused on 208, 222 (α-helix), and 215–217 nm (β-sheets) (Fig. 4a). F475S clearly showed decreased CD signals at 215–217 nm, which suggests that a decreased level of β-sheet formation occurs in the SBD of full-length protein (Fig. 4a), as well as the truncated protein (Fig. 2c). Moreover, F475S variant displayed modified CD signals at 208 and 222 nm compared to WT, which implies that significant conformational changes also occurred in α-helical regions. Considering there are two α-helical rich regions in full-length Ssa1, namely, the NBD and SBDα, we speculate that the conformational changes happened in both or at least one of those regions. To investigate the functional consequences of conformational changes in the α-helix structure of the NBD and the possible functional changes in these mutants, we assessed basal ATPase activity (Fig. 4b). L483W increased ATPase activity at 30 °C (roughly onefold). However, F475S and second-site suppressors all increased intrinsic ATPase activity more significantly (roughly F475S: fivefold; A394V/F475S: threefold; P433S/F475S: fivefold; and V477I/F475S: fivefold). Regarding increased levels of basal ATPase activity by SBD mutants, it has been shown that the SBD acts as a brake on the ATPase activity of the NBD and that the free NBD with the linker domain attached has a higher ATPase activity than full-length Hsp70 [45]. It is thought that the ATPase inhibition from the SBD requires docking of NBD with SBD [9]. Therefore, the elevated basal ATPase rate by SBD mutants suggests that SBD integrity and/or important contacts between NBD and SBD are lost or reduced and prevent inhibition and normal regulation of ATP hydrolysis.

The Hsp70/Hsp40/Hsp104 machinery plays an essential role in prion propagation and thermotolerance. A major function of Hsp70 machinery is refolding proteins that have become denatured due to heat stress. Because SBD disruption induced by substitution, it was predicted that the F475S would be unable to refold a model denatured protein such as luciferase. To assess this, we assayed luciferase refolding activity for F475S and L483W variants. Unsurprisingly, F475S and L483W displayed a deficiency in luciferase refolding (Fig. 4c) in vivo, which correlates with reduced abundance of Ssa1 and indications of reduced levels of substrate-binding affinity (Fig. 4d, e). A reduction of in vivo protein refolding capacity will contribute significantly to the heat shock and prion-impairing phenotypes of these mutants.

In addition, the previous reports showed that Hsp26 impaired [*PSI* ^+^] prion propagation by inhibiting self-templating and preventing conformational rearrangements of molten oligomers in yeast [46] and overexpression of Hsp104 cured [*PSI* ^+^] prion [27, 47]. Therefore, the impairment of [*PSI* ^+^] prion propagation by F475S and L483W may be influenced by the increased affinity of Ssa1 with Hsp26 and/or increased expression these co-chaperones (Fig. 4d, e).

### The NBD-SBD interface regulates prion propagation and the heat-shock response

**Fig. 5:**
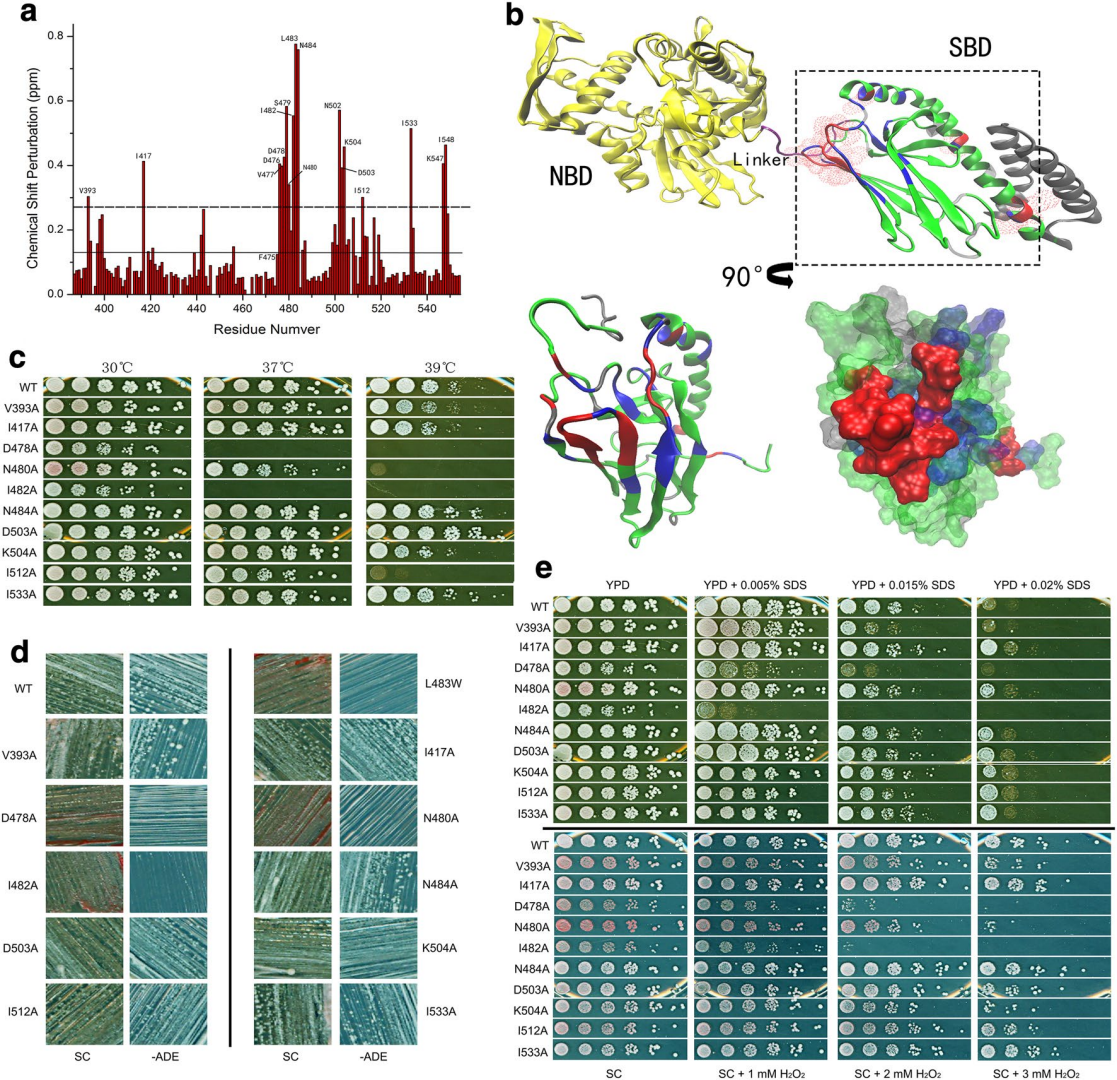
Interface regulates prion propagation and heat-shock response. **a** CSP histogram of L483W compared to WT at 30 °C. The solid line shows the average of the CSPs; the dotted line shows the average plus SD of CSPs. **b** Interface between SBD and NBD. Residues with significant CSPs were mapped onto the full-length DnaK structure (PDB:2KHO); overlay of SBDβ with residues in the SBD model of DnaK (PDB:1BPR) that have significant CSPs. Aspect was rotated by 90° as arrow indicates. The residues with significant CSPs over the average plus SD are in red; the residues with CSPs between the average plus SD and the average are in blue; the residues with CSPs below the average are in green; non-assigned residues are in grey. **c** Growth assay of predicted mutations at elevated temperatures. **d** Assessment of the predicted mutations on [*PSI* ^+^] propagation. [*psi* ^−^] cells were red colonies on YPD and unable to grow on −ADE plates; [*PSI* ^+^] cells were white colonies on YPD and survived on −ADE plates. **e** Growth assay of predicted mutations under other stresses. YPD and SC medium were supplemented with cell-wall damage reagent SDS and oxidative damage reagent H_2_O_2,_ respectively, to achieve required concentrations. Fresh cultures were spotted on those plates after a 1/5 serial dilution and incubated for 2 days at 30 °C

To further investigate the SBDβ conformational changes induced by mutations and heat shock, we utilised NMR for analysis of L483W. We chose this strategy as WT is not sensitive enough to elevated temperature to assess, while conversely, F475S is too disordered to assign residue signals. Figure 5a shows a CSP histogram of L483W compared to WT at 30 °C. There are five regions that are readily identified with significant CSPs: (I) V393 in the linker; (II) I417 in loop 23; (III) D476, V477, D478, S479, N480, I482, L483, and N484 in β6, loop 67 and β7; (IV) N502, D503, and K504 in β8; and (V) I512, I533, K547, and I548 in SBDα. Intriguingly, many residues in those regions have been previously reported as playing critical roles in the SBDβ allosteric transition and inter-domain communication.

Notably, a clear surface is formed by those residues in regions (I), (II), (III), and (IV) (Fig. 5b). Considering the location of this surface, it is located at the interface between SBD and NBD (Fig. 5b). This suggests that the temperature sensitivity and [*PSI* ^+^] phenotypes of L483W strains are related to defects in inter-domain communication in Ssa1. Moreover, region (V) may be involved directly with the regulation of opening and closing the α-helical lid (Fig. 5b). Interestingly, residues of region (V) are far away from L483 and their significant CSPs directly indicate a long-range allosteric effect caused by introduction of the L483W mutation. To further confirm the significant CSPs around residue, 483 are induced by conformational changes, and are not due to the Trp side chain itself, and we performed alanine-scanning mutagenesis on specific residues to decipher the contribution of side-chain hydrogen bonds, hydrophobic interactions, and salt bridges. Importantly, this process resulted in an array of prion and heat-shock phenotypes for individual mutations (Fig. 5c, d; Table 1). V393A is weak [*PSI* ^+^] and heat resistant; I417A, N484A, D503A, K504A, and I533A are [*PSI* ^+^] and heat resistant; I512A is [*PSI* ^+^] but temperature sensitive; D478A, N480A, and I482A are [*psi* ^−^] and temperature sensitive. We also identified D476A as a lethal mutation. In addition, we observed phenotypic differences in response to cell-wall damaging agents (SDS) and oxidative stress (H_2_O_2_) (Fig. 5e; Table 1). Cells expressing these mutations as the only Ssa source has differing abilities in responding to different stresses, suggesting that cells share overlapping pathways to regulate heat shock and other stresses [48], and also highlights the crucial role of these residues in the NBD–SBD interface. Taken together, we show that residues in the linker, L23, β6, L67, β7, and β8 regions of Hsp70, which form the interface between SBD and NBD, play a common and critical role in prion propagation, thermotolerance and general stress responses.

**Table 1:** Phenotypes of the interface mutations

	V393A	I417A	D476A	D478A	N480A	I482A	N484A	D503A	K504A	I512A	I533A
ts	N	N	N/A	Y	Y	Y	N	N	N	Y	N
Prion	Weak	+	N/A	−	−	−	+	+	+	+	+
H_2_O_2_	Y	N	N/A	Y	N	Y	N	N	N	N	N
SDS	Y	N	N/A	Y	Y	Y	N	N	Y	N	N

*ts* temperature sensitive

## Discussion

In this study, we show that a newly identified and highly conserved residue F475, and also the previously reported and partially characterized residue L483, play crucial roles in the structural and functional regulation of cytosolic Hsp70. The substitution mutations F475S and L483W have major impact on Hsp70 function and phenotypes. Structurally, F475 and L483 are located within the β6–β7 region of Hsp70 SBD, which is highly conserved in the Ssa family, and across species also, ranging from *E. coli* DnaK to human Hsp70 and Hsc70 proteins. The F475S variant, which was isolated from a SBD-targeted random mutagenesis strategy, displays deficiencies in [*PSI* ^+^] prion propagation and ability to grow at elevated temperatures. These phenotypes are similar to those exhibited by L483W, but are more pronounced in F475S. From a structural biology point of view, both F475S and L483W cause perturbation of the hydrophobic core, causing destabilisation and accelerating denaturation of this localised region of the SBD in response to elevated temperature. Destabilisation of the β6–β7 region of the SBD ultimately results in altered inter-domain communication between the NBD and SBD through structural changes at the interface of the domains, formed by residues in the linker, and the L23, β6, L67, β7, and β8 regions. The major functional changes in Hsp70 induced by these mutations are ultimately the dysregulation of ATP hydrolysis of the NBD, which results in broad effects such as reduced protein refolding activity, enhanced interactions with co-chaperones such as Hsp104 and Hsp26 but a decreased interaction with substrates such as Sup35.

While F475S and L483W display phenotypic changes in both stress-related growth and prion propagation, it is clear that there is not a linear relationship between these two phenotypes. The previous studies have identified seemingly prion-specific phenotypes in a variety of Hsp70 mutants [34, 40] and it is clear from the array of phenotypes exhibited across the NBD:SBD interface mutants (Fig. 5a–e; Table 1) that a complex relationship exists between Hsp70 functional activity and the phenotypic outcome in stress-related growth and prion propagation. Comparison of temperature sensitivity and prion propagation phenotypes also shows a complex relationship. While L483W and F475S show a similar inability to propagate [*PSI* ^+^], the difference in temperature sensitivity of these mutants highlights an important difference underpinning these two phenotypes. Considering the structural changes occurring in these mutants, of which F475S is the most perturbed, and the phenotypic consequences, it seems reasonable to conclude that prion propagation is more susceptible to fluctuations in Hsp70 functional activity compared to phenotypic changes in temperature sensitivity. The fact that both F475S and L483W are unable to propagate [*PSI* ^+^], but F475S is much more temperature sensitive supports this conclusion.

Insights into the long-range residue interactions and regulatory changes induced by F475S and L483W, in addition to disruption of the hydrophobic core and NBD:SBD interface, can be gleaned from assessment of the second-site suppressors. From a structural aspect, A394V/F475S and V477I/F475S would be predicted to be involved in the allosteric pathway transmitting information from the hydrophobic core to the domain interface, while P433S/F475S would be predicted to exert influence directly in the region of the substrate-binding (Fig. 1a). Interestingly, residue 434 (homologous to 433 in Ssa1) and residue 478 (homologous to 477 in Ssa1) are Ser and Ile in DnaK (Fig. 1a), consistent with the higher optimal growth temperature of *E. coli* cells. It was also found that the side chain of V477 in Ssa1 is located in the hydrophobic core, as is F475 (Fig. 1a), implying that V477I possibly compensates for the disruption caused by the F475S substitution by increasing hydrophobic content in the core.

Both L483W and F475S clearly have a significantly reduced interaction with Sup35 compared to wild-type protein (Fig. 4e), which appears contradictory to the previous genetics and MD results for L483W [40, 43]. However, the most likely explanation is the complexity in identifying appropriate substrate-binding states and possible differences generated through in vitro, in silico, and in vivo studies. While the assay used in this study will assess interaction between Hsp70 and monomeric Sup35, in strains carrying prions, there may well be differential affinities for aggregated and amyloid states of Sup35. Assessing this for L483W in vivo is not possible as strains harbouring this mutant as the sole source of Ssa protein in the cell are [*psi* ^−^].

It is the intricate mechanism of the Hsp70 machine itself that makes it such a versatile protein [10]. We have demonstrated that denaturation of the SBD is one of the causes of cell growth impairment at high temperatures. Previous studies have also reported that Ssa1 mutants (P417L/S) with a temperature sensitivity phenotype were digested more rapidly by proteases in vitro, suggesting structural impairment [41]. Therefore, it is possible that F475S and L483W accelerate the degradation of Ssa1, especially the SBD, which decreases Ssa1 abundance and this in conjunction with additional functional changes results in the phenotypes observed. Considering that F475S causes a much-reduced level of essential Ssa1 in the cell (Fig. 4d), it is surprising that such cells do not exhibit a major growth defect at normal temperatures. This suggests that in unstressed conditions, yeast cells require minimal amounts of cytosolic Hsp70 to carry out its essential functions and/or its functions are compensated for by other chaperones.

Our finding that β6–β7 can act as a location for inducing structural changes and signals within the SBD is in agreement with recent data highlighting the importance of this region [49]. Meanwhile, our data further highlight specific residues in the β6–β7 region of the Hsp70 SBD that play key roles in inter-domain communication. In addition, structural and genetic analysis of these mutants has allowed the characterisation of a potential interface between the NBD and SBD and identification of key residues that influence prion propagation and the stress response, and may play important roles in signal transduction between the domains (Fig. 5). The linker is very important for inter-domain communication and is buried in the NBD cleft after docking [10]. Residues V389, L391, and D393 in DnaK are known to be involved in the interface between NBD and SBD and to regulate inter-domain communication directly [10], suggesting that V393 may have the same effect. P418 in Ssa1 has been reported to alter ATP cycling and inter-domain communication [41]. Residues 414 and 417–420 in DnaK have been identified as an important hinge region in direct contact with the linker [8, 9, 13]. I417 found in the current study may have the same effect as other residues in the loop 2, 3. In region (III) mentioned above, F475S, V477I, and L483W have been shown to have critical structural roles and L484 and D481 in DnaK (homologous to L483 and N480 in Ssa1) regulate signal transduction from the SBD to the NBD [9]. In vivo, neither DnaK–D481A nor DnaK–D481K was able to complement the temperature-sensitive growth defect of the DdnaK52::Cm strain BB1553 [9]. I501 is the lynchpin residue of the hydrophobic core and I501 and S505 are both involved in the SBDβ allosteric network in DnaK [13]. M515I and S545F (close to K547 and I548) have been identified in this study as impairing [*PSI* ^+^] propagation to some degree. I512 found in this study is near to D511E variant (residue number in DnaK), which is located at the interface between SBDβ and the SBDα lid and identified as involved in SBDβ allosteric regulation [13].

The yeast Hsp70 model system provides the ideal environment for dissecting the intricacies of Hsp70 regulation and how changes in function relate to phenotypic changes in the heat-shock response and prion propagation. The critical β6–β7 region and the potential NBD:SBD interface we have identified are therapeutic targets which can be used to design specific inhibitors or modifiers of Hsp70 function.

## Materials and methods

### Plasmids and yeast strains

The *SSA1* gene and required mutations were constructed in pC210 plasmid [30] using a site-directed mutagenesis kit (Agilent) and primers used are shown in Table S1. Plasmids were transformed into strain G402 (*MATa ade2*-*1 SUQ5 kar1*-*1his3 leu2 lys2 trp1 ura3 ssa1::KanMX, ssa2::HIS3, ssa3::TRP1, ssa4::URA3*-*1f/pRDW10*) [50], and colonies re-streaked onto plates containing 5-fluoroorotic acid (5-FOA) to select against cells harbouring the *URA3* plasmid containing wide-type Ssa1.

### Random mutagenesis

Using random mutagenesis to isolate Ssa1 mutations that alter prion propagation has been described before [40]. In this study, we specifically targeted the SBD to generate new mutations impairing prion propagation. The pJ120 vector [34] was incubated in hydroxylamine for 1 h at 70 °C. The SBD was PCR amplified from this plasmid library targeted primers containing appropriate restriction sites at the ends. The PCR products were sub-cloned into a *SexA*1 and *Sph*1 digested pJ120. This process results in Ssa1-containing plasmids with potentially mutated regions solely within the SBD. This pJ120 ligated library was transformed and amplified in *E. coli*. The plasmid library was isolated from *E. coli* and transformed into G402 and replica-plated onto 5-FOA medium. Any red or clearly pink colonies had plasmids isolated and re-transformed back into G402 to confirm their inability to propagate [*PSI* ^+^]. This process identified F475S as the most extreme Ssa1 mutant in terms of impairing prion propagation in yeast. We identified second-site suppressors of F475S by randomly mutagenizing F475S containing plasmid and selecting for reversal of phenotypes.

### Yeast growth assay

Yeast strains were cultured in 5 ml YPD or SC with appropriate selection at 30 °C overnight. The following morning, the yeast cultures were diluted into 6 ml fresh media at an OD_600_ of 0.2 and grown until the OD_600_ reached 0.5. A 1/5 serial dilution was performed in a 96-well plate and then replicated onto appropriate media. Plates were incubated at 30 °C or elevated temperatures (37 or 39 °C) for 2 days as required.

### Protein purification

Full-length Ssa1 (residues 2–642), truncated Ssa1 (residues 382–554) [51] and corresponding mutations were constructed in the pET28a-Smt3 vector containing 6× His and Smt3 tags [52]. The plasmid was transformed into BL21-CodonPlus (DE3)-RIL competent cells and induced by IPTG in 2YT medium. For NMR, M9 minimal medium containing ^15^N–NH_4_Cl or ^15^N–NH_4_Cl/^13^C–glucose was used. Full-length Ssa1 and mutations were first purified on an Ni affinity column (GE Healthcare) in 50 mM Tris buffer (pH 7.5), 300 mM NaCl, and 3 mM β-mercaptoethanol. Protein product was then incubated with Ulp1 followed by a second Ni affinity column purification step to remove the 6× His-Smt3 tag, Ulp1, and un-cleaved protein. The flow through was collected and further purified by gel filtration chromatography (Superdex 200, GE Healthcare) in 50 mM Tris buffer (pH 7.5), 100 mM KCl, and 5 mM MgCl_2_. The truncation mutants were similarly purified with some modifications to buffers. For Ni affinity purification, 50 mM Tris buffer (pH 8.0) and 200 mM NaCl were used. Gel filtration chromatography was performed in 50 mM Na–phosphate buffer (pH 7.0) with 50 mM NaCl (Superdex 75, GE Healthcare).

### Circular dichroism spectroscopy

The CD spectra of full-length Ssa1 and mutations were obtained in 50 mM Tris buffer (pH 7.5), 100 mM KCl, and 5 mM MgCl_2_ at a protein concentration of 3 μM using a Chirascan Plus CD spectrometer (Applied Photophysics, UK). Spectra were measured from 200 to 260 nm in a 10 mm path-length thermostat-controlled quartz cuvette. The temperature was controlled using a Peltier device. The truncation mutants, at a protein concentration of 10 μM, were measured in 50 mM Na-phosphate buffer (pH 7.0) with 50 mM NaCl.

### Size exclusion chromatography (SEC) and MALDI-TOF

The pattern of SBD truncation mutants was investigated by SEC (Superdex 75, 24 ml GE Healthcare) in 50 mM Na-phosphate buffer (pH 7.0) with 50 mM NaCl at room temperature. To detect the molecular weight of the F475S truncation mutant, the protein was passed through a C18 ziptip to remove salt and then mixed with SA (Sinapic acid) matrix. MALDI-TOF was performed on a MALDI-TOF AXIMA-CFR Plus (KRATOS Analytical, Shimadzu Corporation, Japan).

### ATPase assay

The ATPase activity was measured based on colorimetric determination of inorganic Na-phosphate using malachite green as previous described [53] with minor modifications. Briefly, stock solutions of malachite green (0.081% w/v), polyvinyl alcohol (1.15% w/v), and ammonium heptamolybdate tetrahydrate (2.85% w/v in 3 M HCl) were prepared, and mixed in the ratio of 1:1:1 to prepare the malachite green reagent. Before assay, 10 μl of 2 μM Ssa1 (50 mM Tris buffer pH 7.5, 100 mM KCl, 5 mM MgCl_2_ and 1 mM DTT), and 10 μl of 2 mM ATP were mixed into each well of a 96-well plate, and then incubated at 30 °C for 5 h. After incubation, 80 μl of malachite green reagent was added to each well. Immediately following the malachite green reagent, 10 μl 34% sodium citrate was added to stop the non-enzymatic hydrolysis of ATP. The samples were mixed thoroughly and incubated at 30 °C for 30 min before measuring the OD_620_ on a SpectraMax M3e (Molecular Devices, USA). Intrinsic ATP hydrolysis was accounted for by subtracting the signal measure for ATP incubated in the absence of Ssa1.

### Nuclear magnetic resonance (NMR) spectroscopy

Nuclear magnetic resonance spectroscopy was employed in this study to investigate conformational changes of the SBD induced by mutants and heat shock. For acquisition of 2D ^1^H–^15^N HSQC spectra, NMR samples were prepared in 50 mM Na-phosphate buffer (pH 7.0), 50 mM NaCl, 0.02% NaN_3_, 5 mM EDTA, 10 mM DTT, and 10% D_2_O. NMR experiments were performed at 30, 37, and 39 °C on a Varian INOVA 600 MHz spectrometer equipped with a triple-resonance cryo probe. To increase the signals in the 3D ^1^H–^15^N HSQC spectra of the L483W truncation mutant, NMR samples were prepared in 20 mM Na-phosphate buffer (pH 7.0) containing 0.02% NaN_3_, 5 mM EDTA, 10 mM DTT and 10% D_2_O. Spectra were collected at 35 °C. After collection, the sample was titrated back to 50 mM Na-phosphate buffer (pH 7.0) and 50 mM NaCl to allow direct comparison with the buffer used as WT sample. In addition, during titrations, 2D spectra were collected at 35 and 30 °C to collate chemical shifts caused by changes in temperature. Other details were as previously described [51].

### Luciferase refolding assay

The luciferase assay was carried out as previously described [50]. Briefly, the G402 yeast strains containing pDCM90 were cultured in 5 ml selective medium without uracil at 30 °C overnight. Then, cultures were diluted to OD_600_ of 0.2 into same the medium and incubated at 37 °C shaking for 30 min to induce expression of heat-shock proteins. After culturing, the cellular luciferase activity of each strain was measured by immediately adding 10 μl of decanal (Sigma) to 200 μl of culture in an FB12 Luminometer (Berthold Detection Systems) as a reading for 100% activity. Cells were then transferred into a 45 °C shaking incubator for 1 h. During this 1 h heat shock, cyclohexamide (Sigma) was added after 50 min at a concentration of 10 μg/ml. After thermal denaturation at 45 °C, cellular luciferase activity was measured as the starting point (*t* = 0). Cultures were shifted to 25 °C at intervals of 15 min for a duration of 1 h. Luciferase recovery ratio was calculated basing on staring point (*t* = 0) and ending point after 1 h recovery as a percentage of the 100% activity.

### Protein extraction from yeast

Yeast strains were cultured overnight in 5 ml YPD or selective media at 30 °C. The following morning, cells were diluted in 25 ml fresh same media to an OD_600_ of 0.2 and incubated until an OD_600_ of 0.6–0.8 was reached. Cells were harvested by centrifugation at 4 °C (5 min, 2500 rpm) and pellets were washed with distilled water. Pellets were resuspended in yeast cell lysis reagent (Sigma C4482) complemented with 10 mM DTT (without DTT for pull down) and protease inhibitor cocktail (Sigma P8215). Glass beads of 0.5 mm were used to aid cell lysis using mini-beater (Biospec products). Supernatants were transferred to pre-chilled 1.5 ml microfuge tubes and centrifuged (10 min, 13,000 rpm) to remove any cell fragments.

### Pull-down assay

Anti-Flag M2 Magnetic Beads (Sigma M8823) was used to purify Flag-tagged Ssa1 in yeast cells. Protein extraction of 300 μg was incubated with 50 μl magnetic beads gently on a rotator at 4 °C overnight. Magnetic beads were collected by magnetic separator and washed with ten volumes of TBS (50 mM Tris–HCl buffer, 150 mM NaCl, pH 7.5) four times. Three volumes of 3× Flag peptide of 150 ng/μl were added and the samples were incubated on a rotator for 1 h at 4 °C. Supernatants were transferred to pre-chilled 1.5 ml microfuge tubes and centrifuged (10 min, 13,000 rpm). Then, 25 μl was analysed by western blotting.

### Molecular dynamics (MD) simulation

Molecular dynamics simulations were performed using the GROMACS 4.5.7 and 4.6 package [54] as described previously [43]. The homologous Hsp70 solution structure of Ssa1, DnaK SBD with peptide NRLLTG (PDB code: 1Q5L) [55] was used as wild type. Appropriate mutations were constructed using Swiss-Pdb Viewer [56] based on wild type. Simulations were carried out over a 20 ns period at 300, 310 and 312 K, pH 7.0 and 1 bar pressure. The coordinate trajectories were saved for subsequent data analysis.

## Electronic supplementary material

Below is the link to the electronic supplementary material.

Supplementary material 1 (DOCX 233 kb)
