# Supplementary material for: The β6/β7 region of the Hsp70 substrate-binding domain mediates heat-shock response and prion propagation
Source: Cell Mol Life Sci. 2017 Nov 9;75(8):1445–59. doi: 10.1007/s00018-017-2698-3 (PMC5852193; doi:10.1007/s00018-017-2698-3)
Supplement: Supplementary file 1 — Supplementary material 1 (DOCX 233 kb) [file 18_2017_2698_MOESM1_ESM.docx]

**Supporting information**

Fig. S1


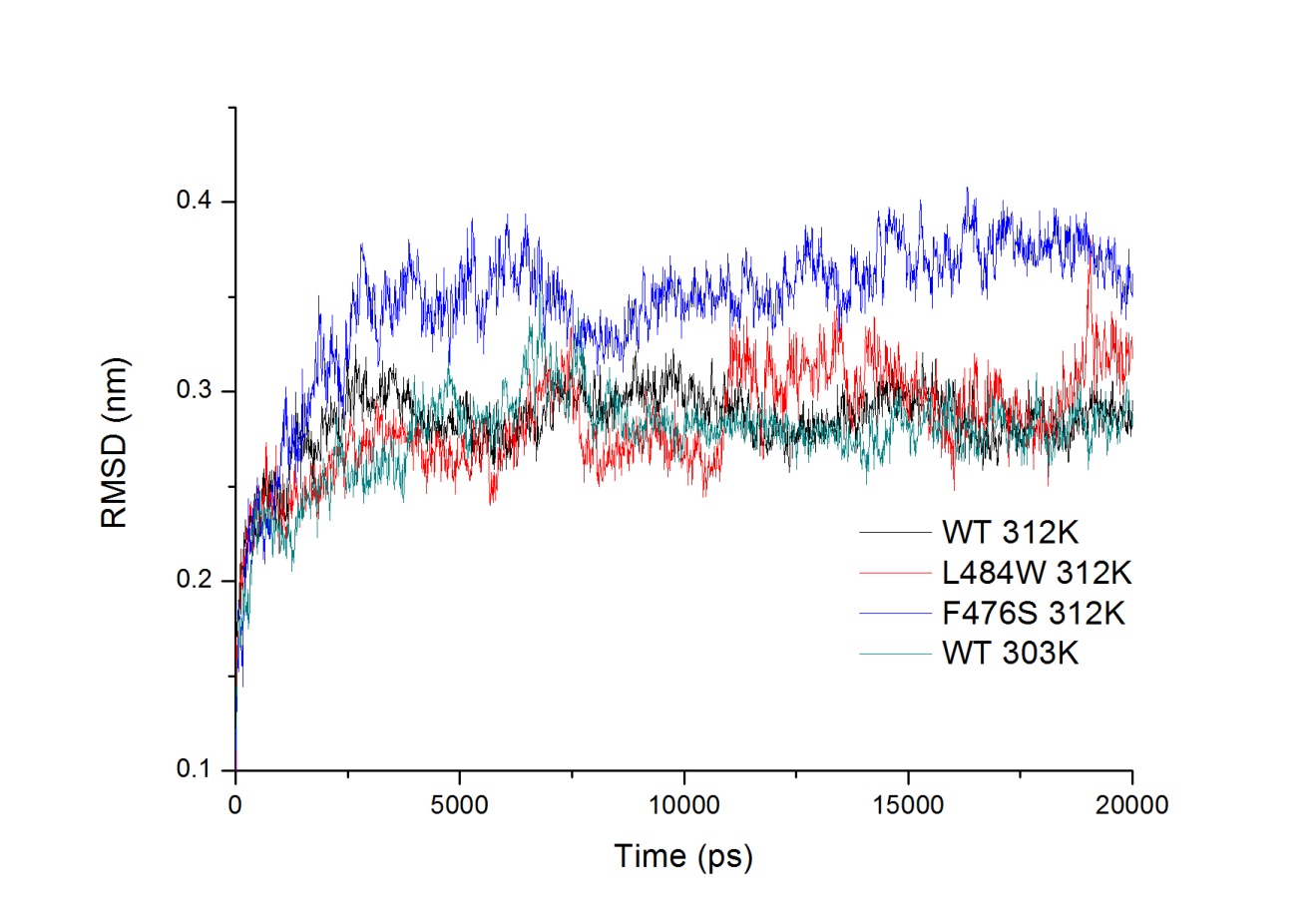


Fig. S1. RMSDs as a function of the simulation time. RMSDs were calculated using the g_rms program based on the Cα atoms of the amino acid backbone of the DnaK protein.

Table S1. List of Primers

| V435I | F 5’ CAGGTATCTTGATTCAAGTCTTTGAAGGTGAAAG 3’ |
| --- | --- |
| V435I | R 5’ TCAAGATACCTGGTTGGTTATCAGCATAAGTGG 3’ |
| R444K | F 5’ GGTGAAAAAGCCAAGACTAAGGACAACAACT 3’ |
| R444K | R 5’ TGGCTTTTTCACCTTCAAAGACTTGAATCAA 3’ |
| A394V | F 5’ TGTCGTTCCATTATCCTTGGGTATTGAAACT 3’ |
| A394V | R 5’ ATGGAACGACATCCAACAACAATAGATCTTGA 3’ |
| P433S | F 5’ ACCAATCAGGTGTCTTGATTCAAGTCTTTG 3’ |
| P433S | R 5’ ACACCTGATTGGTTATCAGCATAAGTGGAA 3’ |
| V477I | F 5’ CGATATCGACTCTAACGGTATTTTGAATGTTTCC 3’ |
| V477I | R 5’ GTCGATATCGGAAGTGACTTCAATTTGTGGG 3’ |
| F475S | F 5’ AGTCACTTCCGATGTCGACTCTAACGGTATTTTG 3’ |
| F475S | R 5’ GTCGACATCGGAAGTGACTTCAATTTGTGGG 3’ |
| F475W | F 5' TTGAAGTCACTTGGGATGTCGACTCTAACGGTATT 3' |
| F475W | R 5' GTCGACATCCCAAGTGACTTCAATTTGTGGGACAC 3' |
| F475Y | F 5' TTGAAGTCACTTACGATGTCGACTCTAACGGTATT 3' |
| F475Y | R 5' GTCGACATCGTAAGTGACTTCAATTTGTGGGA 3' |
| F475C | F 5' TTGAAGTCACTTGCGATGTCGACTCTAACGGTATT 3' |
| F475C | R 5' GTCGACATCGCAAGTGACTTCAATTTGTGGGA 3' |
| F475A | F 5' TTGAAGTCACTGCCGATGTCGACTCTAACGGTATT 3' |
| F475A | R 5' GTCGACATCGGCAGTGACTTCAATTTGTGGGA 3' |
| L483W | S 5’ GTATTTGGAATGTTTCCGCCGTCGAAAAGGGT 3’ |
| L483W | A 5’ CGGAAACATTCCAAATACCGTTAGAGTCGACATCG 3’ |
| L483A | S 5’ GTATTGCGAATGTTTCCGCCGTCGAAAAGGGT 3’ |
| L483A | A 5’ CGGAAACATTCGCAATACCGTTAGAGTCGACATCG 3’ |
| L483H | S 5’ GGTATTCATAATGTTTCCGCCGTCGAAAAGGGT 3’ |
| L483H | A 5’ GCGGAAACATTATGAATACCGTTAGAGTCGACATCG 3’ |
| V393A | F 5’ GTTGTTGGATGCCGCTCCATTATCCTTGGGTATTG 3’ |
| V393A | R 5’ GATAATGGAGCGGCATCCAACAACAATAGATCTTGAGTCT 3’ |
| I417A | F 5’ ACTCTACCGCTCCAACAAAGAAGTCCGAGATCTTTTCC 3’ |
| I417A | R 5’ CTTTGTTGGAGCGGTAGAGTTTCTTGGAATCAACTTGG 3’ |
| D476A | F 5’ GAAGTCACTTTCGCTGTCGACTCTAACGGTATTTTGAATGT 3’ |
| D476A | R 5’ GAGTCGACAGCGAAAGTGACTTCAATTTGTGGGACAC 3’ |
| D478A | F 5’ CACTTTCGATGTCGCCTCTAACGGTATTTTGAATGTTTCC 3’ |
| D478A | R 5’ CCGTTAGAGGCGACATCGAAAGTGACTTCAATTTGTGGGA 3’ |
| N480A | F 5’ ATGTCGACTCTGCCGGTATTTTGAATGTTTCCGCCG 3’ |
| N480A | R 5’ TCAAAATACCGGCAGAGTCGACATCGAAAGTGACTTC 3’ |
| I482A | F 5’ CTCTAACGGTGCTTTGAATGTTTCCGCCGTCGA 3’ |
| I482A | R 5’ GGAAACATTCAAAGCACCGTTAGAGTCGACATCGAAAG 3’ |
| N484A | F 5’ CTCTAACGGTATTTTGGCTGTTTCCGCCGTCGA 3’ |
| N484A | R 5’ GGCGGAAACAGCCAAAATACCGTTAGAGTCGACATC 3’ |
| D503A | F 5’ CTATTACCAACGCCAAGGGTAGATTGTCCAAGGAAGATATC 3’ |
| D503A | R 5’ GGACAATCTACCCTTGGCGTTGGTAATAGTGATCTTGTTAG 3’ |
| K504A | F 5’ CTATTACCAACGACGCGGGTAGATTGTCCAAGGAAGATATC 3’ |
| K504A | R 5’ GGACAATCTACCCGCGTCGTTGGTAATAGTGATCTTGTTAG 3’ |
| I512A | F 5’ CCAAGGAAGATGCCGAAAAGATGGTTGCTGAAGC 3’ |
| I512A | R 5’ CCATCTTTTCGGCATCTTCCTTGGACAATCTACCCTT 3’ |
| I533A | F 5’ GAATCTCAAAGAGCTGCTTCCAAGAACCAATTGGAATCC 3’ |
| I533A | R 5’ TCTTGGAAGCAGCTCTTTGAGATTCCTTTTCATCTTCTTC 3’ |
